# SHMT2 modulates the transcriptome and metabolism profiles to promote the tumor phenotypes of bladder cancer HT-1376 cells

**DOI:** 10.3389/fgene.2025.1694089

**Published:** 2025-11-20

**Authors:** Xiaobo Guo, Gang Li

**Affiliations:** Department of Urology, Heji Hospital Affiliated with Changzhi Medical College, Changzhi, Shanxi, China

**Keywords:** bladder cancer, SHMT2, transcriptome, metabolism, cellular phenotypes

## Abstract

**Introduction:**

Bladder cancer (BLCA) is a common malignant tumor of the urinary system. The development and progression of BLCA are controlled by multiple regulatory molecules, which have not been widely investigated.

**Methods:**

In this study, we explored the functions and downstream targets of serine hydroxy methyltransferase 2 (SHMT2) by silencing its expression using small interference RNA (siSHMT2) in HT-1376 cells. Whole transcriptome and metabolism profiles were deeply analyzed to identify the molecular targets of SHMT2.

**Results:**

We found that siSHMT2 significantly reduced the proliferation rate and altered the cell cycle stages of HT-1376 cells. Moreover, siSHMT2 can modulate the expression of hundreds of genes (DEGs), including 126 upregulated and 106 downregulated DEGs. We then found that the most significant DEGs were tightly associated with progression of cancers, including *PTMA*, *HNRNPR*, *RAPH1*, *TRAF3IP1*, *CNBP*, and *PRR15*. At the same time, the alternative splicing profile was also regulated by siSHMT2, including the skipped exon as the dominant AS types. Then we confirmed the changed expression levels of *PTMA*, *HNRNPR*, *RAPH1*, and *CNBP*, and AS level of *MDM2* by RT-qPCR. Finally, we identified the differential metabolites (DMs), and found the metabolism profile was significantly regulated by siSHMT2. Besides the purine metabolism, we observed that valine, leucine and isoleucine biosynthesis and degradation, TCA cycle, and propanoate metabolism were among the top pathways of DMs.

**Discussion:**

In summary, we highlight the important roles of SHMT2 in HT-1376 cells and identified its downstream molecular targets, which are associated with the development of BLCA and can be used as therapeutic targets of BLCA in future.

## Introduction

Bladder cancer (BLCA), one of the malignant tumors occurred in the urinary system, was diagnosed with 0.61 million new cases and 0.22 million deaths in the world in 2022 (Bray et al., 2024), indicating the emergency to develop efficient therapeutic methods to treat BLCA. The development of BLCA can be divided into two distinct pathways that have unique pathological features and different molecular characteristics (Sanli et al., 2017). Several factors, including genes and pathways, have been summarized to be associated with and as the key drivers of the development of BLCA (Knowles and Hurst, 2015). Among these factors, the disorder of metabolism is one of the key features of BLCA development and deeply influences the proliferation and metastasis of BLCA cells (Massari et al., 2016). One recent study demonstrated that glucose metabolism can be treated as a therapeutic target for BLCA by enhancing the efficacy of immunotherapy and eliminating malignant cells (Afonso et al., 2020). The metabolic reprogramming of tumor cells can result in the activation of inflammasome and dendritic cell maturation, thus optimizing bladder cancer management (Oresta et al., 2021). In this way, it is important to decipher the driving molecules that are essential for the metabolic process of BLCA.

In BLCA, we have observed that the serine hydroxymethyltransferase-2 (SHMT2) was dysregulated and associated with the poor prognosis of BLCA patients. SHMT2 is an enzyme that can convert serine into glycine and a tetrahydrofolate-bound one-carbon unit, promoting tumor growth by supporting thymidine synthesis and purine synthesis (Zeng et al., 2021; Yang et al., 2018). Moreover, SHMT2 affected the growth, migration, and apoptosis of BLCA cells *in vitro* (Su et al., 2024). For mechanism, it is reported that SHMT2 can inhibit the mitochondrial-mediated apoptosis via the intrinsic signaling pathway in BLCA cells, thus promoting the cell viability and progression of BLCA (Zhang et al., 2022). At the same time, SHMT2 can significantly affect the overall survival and disease-free survival time of BLCA patients, and change the proliferation and apoptosis levels of BLCA cells (Zhang and Yang, 2021). At the same time, the research of SHMT2 in bladder cancer is not very in-depth, and its downstream targets have not been well revealed.

In this study, to further decipher the cellular and molecular functions of SHMT2 in BLCA, we performed an integrated multi-omics experiments to explore how SHMT2 affects the cellular phenotypes and its downstream molecular targets. In brief, we knocked down SHMT2 expression by small interfering RNA (siRNA) in HT-1376 cells, and evaluated its influence on cell cycle, cell proliferation, and other phenotypes. More importantly, we performed whole transcriptome sequencing (RNA-seq) and metabolic profile to investigate the dysregulated RNA and metabolic molecules induced by SHMT2 in HT-1376 cells. In summary, our study provides a comprehensive profile for the targets of SHMT2 in BLCA cells, and the identified molecules can be treated as potential therapeutic targets for BLCA in future.

## Materials and methods

### Cell culture and small interfering RNA transfection

The HT-1376 cells were purchased from Suncell (SNLM-326, Suncell, Wuhan, China). The siRNA duplexes were purchased from Gemma (Suzhou, China). Non-targeting control siRNA (siNC) sequences were 5ʹ-UUC​UCC​GAA​CGU​GUC​ACG​UTT-3ʹ (sense). Four siRNAs targeting SHMT2 (siSHMT2) were designed and one siRNA was finally chosen with sequences 5ʹ-CGA​AUC​AAC​UUU​GCC​GUG​UTT-3ʹ (sense).

The HT-1376 cells were cultured at 37 °C with 5% CO_2_ in Leibovitz’s L-15 with 10% fetal bovine serum (FBS), 100 μg/mL streptomycin, 100 U/mL penicillin. The siRNA transfection into the cells was performed using Lipofectamine™ 2000 transfection reagent (52887, Invitrogen, Carlsbad, CA, USA) according to the manufacturer’s protocol. We collected the transfected cells after 24 h for following experiments.

### Cell proliferation and cell cycle experiments

CCK8 testing method and kit (HYCCK8-500T, HYCEZMBIO) were used to assess the proliferation level of HT-1376 cells. After siRNA transfection for 24h, the HT-1376 cells were added with 10 μL CCK8 solution and cultured for 4 h at 37 °C. Then the cells were measured with the optical density (OD) by Microplate Reader (ELX800, Biotek, Winooski, VT, USA) at an absorbance of 450 nm, and finally assessed the proliferation level. For cell cycle experiment, the cell suspension was added to 3.5 mL of pre-cooled 80% ethanol and fixed overnight at 4 °C, and then centrifuged at 2000 rpm for 5 min. We joined 500 μL PI/RNase staining buffer resuspend cells, passed through 200 mesh nylon sieve, and prepared a single-cell suspension. Then the cells were incubated at 4 °C in dark for 30 min. We detected red fluorescence and light scattering at an excitation wavelength of 488 nm using a flow cytometer (FACSVerse, BD, USA). Analysis software is used for cell DNA content analysis and light scattering analysis to evaluate cell cycle characteristics.

### RNA-seq library construction and analysis

For each sample, we took 1.5 μg of qualified total RNA for RNA-seq library preparation. Firstly, VAHTS mRNA capture beads 2.0 (N403, Vazyme, Nanjing, China) was used to capture and purify total RNA. Then, VAHTS Universal V8 RNA seq Library Prep Kit for Illumina (NR605, Vazyme) was used to prepare RNA-seq chain specific libraries through fragmentation, first strand synthesis, second strand synthesis, end repair, linker ligation, amplification, purification, and other steps. Finally, we used Qubit 4.0 (ThermoFisher, Waltham, Massachusetts, USA) for library concentration quantification and stored the libraries at −80 °C in a refrigerator. The RNA-seq libraries was sequenced using NovaSeq X plus (Illumina, San Diego, California, USA) and PE150 mode for high-throughput sequencing.

After obtaining the raw RNA-seq reads, the quality of raw reads was checked using FastQC software (version 0.11.9, https://www.bioinformatics.babraham.ac.uk/projects/fastqc/). Raw paired-end reads were subjected to trimming and quality control using fastp (version 0.23.4) with default parameters (Chen et al., 2018). The quality filtered reads were then aligned separately to the reference genome via HISAT2 software (version 2.2.1) (Kim et al., 2019). Transcriptome changes were analyzed by DESeq2 package (version 1.42.1) and curated by manual and automated methods (Love et al., 2014). We finally selected differentially expressed genes (DEGs) with criteria fold change >1.5 and adjusted *p*-value <0.05. For alternative splicing (AS) analysis, we used rMATS software (Shen et al., 2014) to detect the changed AS pattern by siSHMT2 using default parameters. Finally, ratio difference >5% and false discovery rate (FDR) < 0.05 were set as the threshold of significant AS events. Gene function and pathway enrichment of GO and KEGG were performed using clusterProfiler package (version 4.10.1) (Wu et al., 2021).

### Reverse transcription and quantitative polymerase chain reaction (RT-qPCR)

After reverse transcription (described above), the qPCR step was performed on the ABI QuantStudio 5, followed by denaturing at 95 °C for 10 min, 40 cycles of denaturing at 95 °C for 15 s and annealing and extension at 60 °C for 1 min. Each sample had three technical replicates. The relative level of each transcript was then normalized to GAPDH (glyceraldehyde-3-phosphate dehydrogenase) and calculated using 2^−ΔΔCT^ method (Livak and Schmittgen, 2001). Comparisons were performed with the two-way ANOVA or the paired Student’s t-test by using GraphPad Prism software (Version number8.0, San Diego, CA). The primer sequences for PCR were shown in Supplementary Table S1.

### Metabolism experiment and analysis

The HT-1376 cells were lysed for metabolite extraction by adding 1 mL of 80% methanol solution (containing a mixture of internal standards) to the cell culture dish. After vacuum freeze-drying, resuspend in 100 μL of 10% methanol/90% water solution, vortex for 30 s, sonicate for 1 min, centrifuge at 14000 *g*, 4 °C for 10 min, transfer the supernatant to a sample vial for mass spectrometry analysis. Metabolites were separated using a Waters ACQUITY BEH C18 Column (1.7 µm × 2.1 mm x100 mm) on a Vanquish Flex UPLC equipped with a refrigerated autosampler (10 °C) and column heater (40 °C). Quality control samples (PQC) are prepared by mixing sample extracts to analyze the reproducibility of samples under the same processing conditions.

Utilizing the Human Metabolome Database (HMDB) and the Kyoto Encyclopedia of Genes and Genomes (KEGG) pathway database, all detected metabolites are annotated, functionally defined, and classified. Visualization is performed using the ggplot2 (version 3.4.4) package. Based on the log2 transformed raw data, differential metabolites are identified using both univariate and multivariate statistical analysis. Utilizing the KEGG pathway database, Fisher’s exact test is employed to perform KEGG pathway enrichment analysis on the identified differential metabolites. Based on all identified metabolites and the KEGG pathway database, Metabolite Set Enrichment Analysis (MSEA) is conducted using the R package corto (version 1.2.4), with the parameter np (Number of Permutations) set to 500.

### Statistical analysis

The comparison among different groups was analyzed by Student’s t-test for two groups and one-way ANOVA test for multiple groups.

## Results

**FIGURE 1:**
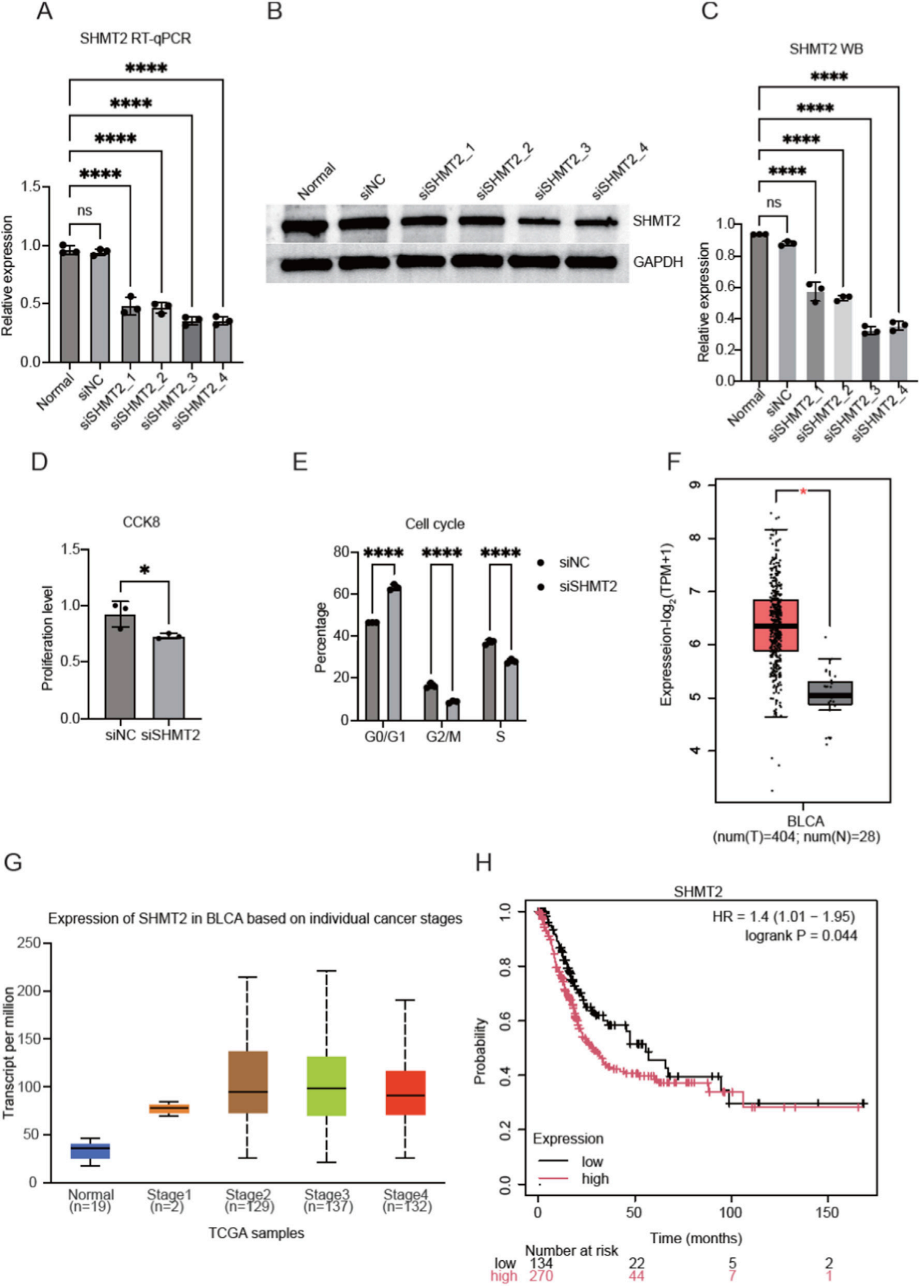
SHMT2 knockdown significantly altered the phenotypes of HT-1376 cells. **(A)** Bar plot showing the RT-qPCR results for SHMT2 in normal, siNC, and four siSHMT2 groups. Ns, non-significant; **** *p*-value <0.0001; N = 3. **(B)** Gel presentation showing the Western blot results for normal, siNC, and four siSHMT2 groups. **(C)** Bar plot showing the quantitative results of Western blot results for the six groups. Ns, non-significant; **** *p*-value <0.0001; N = 3. **(D)** Bar plot showing the decreased proliferation level of siSHMT2-treated HT-1376 cells by CCK8 assay. * *p*-value <0.05; N = 3. **(E)** Bar plot showing the altered percentage of cells in different cell cycle stages by siSHMT2-treatment. **** *p*-value <0.0001; N = 3. **(F)** Box plot showing the differential expression of SHMT2 in BLCA patients. * indicated differential expression. **(G)** The same as **(F)** but for the expression levels of SHMT2 in different BLCA stages. **(H)** Line plot showing the overall survival probability for BLCA patients divided by the expression level of SHMT2.

In this study, we silenced the expression of SHMT2 by small interfering RNA (siRNA) to explore its functions and downstream targets in HT-1376 cells. By conducting the RT-qPCR experiment, we found the four siRNAs can significantly repress the RNA levels of SHMT2 (Figure 1A), indicating the successful knockdown of SHMT2. We then performed WB experiment to confirm the decreased protein level of SHMT2, and found a consistent lower expression level of SHMT2 (Figure 1B; Supplementary Figure S1A). The quantitative result of WB demonstrated a significant decrease of SHMT2 (Figure 1C). Finally, we chose the third siRNA (siSHMT2_3) for following experiments as it showed the lowest level of SHMT2 (Figure 1C). To further investigate the cellular functions of SHMT2 in HT-1376 cells, we performed CCK8 assay to evaluate the proliferation rate, and found that siSHMT2 significantly decreased the proliferation rate of HT-1376 cells (Figure 1D). Meanwhile, cell cycle assay by flow cytometry was conducted to confirm the influence of SHMT2 on cell proliferation. The results demonstrated that siSHMT2 significantly changed the ratio of cells in G0/G1, G2/M, and S stages (Figure 1E). Meanwhile, we found that SHMT2 showed higher expression level in bladder urothelial carcinoma (BLCA) tumor samples compared with adjacent normal samples (Figure 1F). Then we checked the expression level of SHMT2 in different cancer stages of BLCA patients, and found that SHMT2 showed significant increase in all stages (Figure 1G). Prognosis analysis for BLCA patients demonstrated that higher expression level of SHMT2 was associated with worse survival time (Figure 1H). In summary, the above results all together showed that siSHMT2 has profound influence on the fate of HT-1376 cells and BLCA patients, implying its important functions in BLCA.

### SHMT2 slightly modulated the expression profile of HT-1376 cells

**FIGURE 2:**
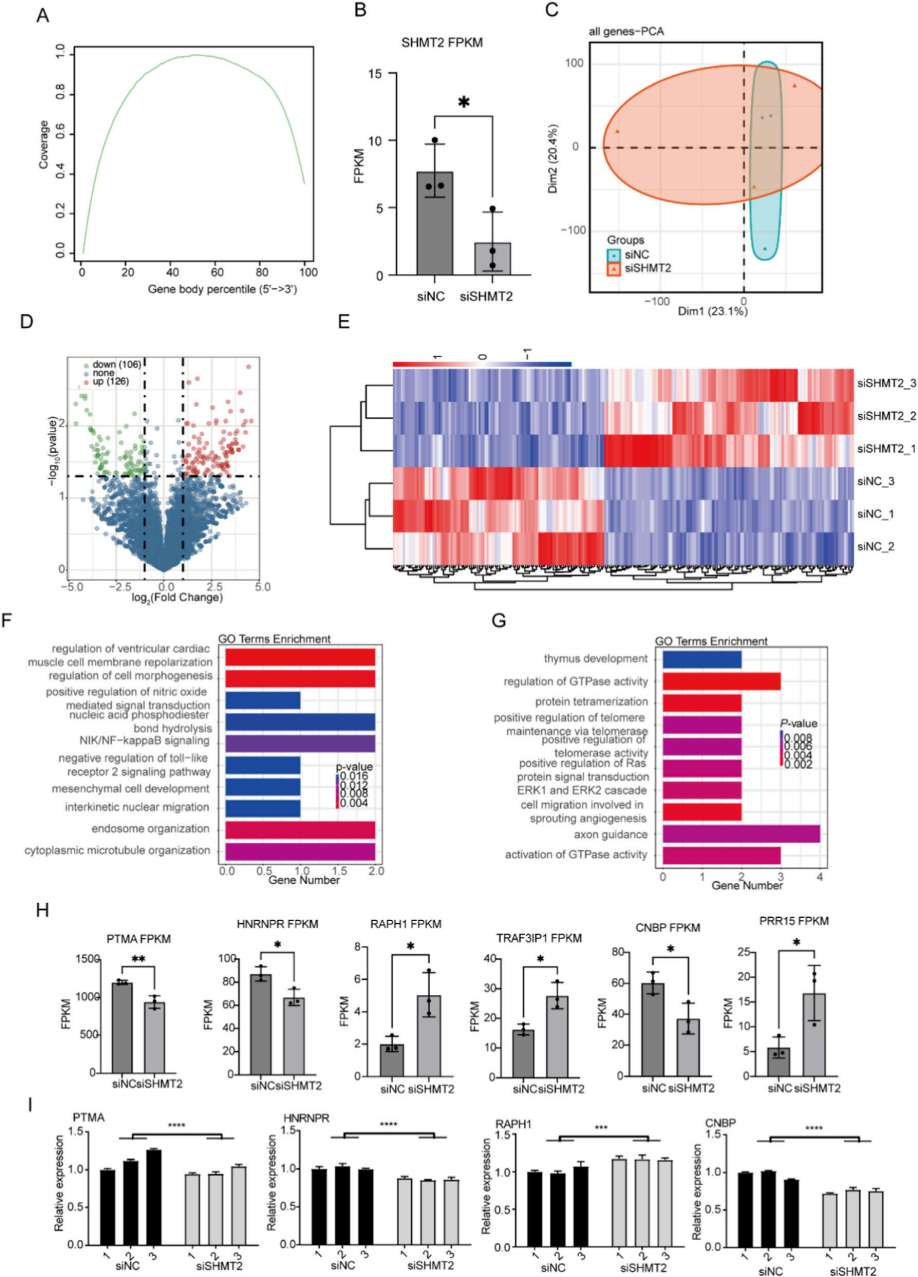
SHMT2 slightly modulated the expression profile of HT-1376 cells **(A)** Line plot presenting the reads distribution on the mRNAs after normalized length. **(B)** Bar plot showing the significantly downregulation of SHMT2 in siSHMT2 samples by RNA-seq. * *p*-value <0.05; N = 3. **(C)** PCA results showing the sample distribution according to the top two principal component. **(D)** Volcano plot showing the log_2_FC and log_10_ *P*-value for all detected genes and DEGs (red and green points for up and down DEGs, respectively). **(E)** Heatmap showing the expression pattern of all detected DEGs. **(F)** Bar plot showing the top enriched GO BP pathways for down DEGs. **(G)** The same as **(F)** but for the up DEGs. **(H)** Bar plot showing the expression pattern of six top DEGs. ** *p*-value <0.01; * *p*-value <0.05; N = 3. **(I)** Bar plot showing the expression levels of for DEGs by RT-qPCR. **** *p*-value <0.0001; *** *p*-value <0.001; N = 3.

To further identify how SHMT2 affect the development and progression of BLCA, we performed RNA-seq experiment based on the siSHMT2 and siNC cells with biological replicates. We obtained about 60 million sequenced reads per sample, and then aligned the quality-filtered reads onto the human genome (GRCh38 version) using the HISAT2 software (Kim et al., 2019), and obtained high quality aligning result (Supplementary Table S2). We found most of the aligned reads came from the transcribed regions, including coding sequence (CDS) and three prime untranslated-region (3′UTR) as the two dominant regions (Figure 2A). FPKM value of SHMT2 also showed significant decrease in siSHMT2 samples (Figure 2B), indicating the successful knockdown of SHMT2. The PCA result demonstrated that the siSHMT2 and siNC groups could not be clearly separated based on the top two principles (Figure 2C), indicating that siSHMT2 did not globally change the expression profile of HT-1376 cells. To further verify that the expression pattern was slightly modulated by siSHMT2, we performed differentially expressed genes (DEGs) analysis. With fold change >1.5 and *p*-value <0.05 as criteria, we identified 232 DEGs, including 126 upregulated DEGs and 106 downregulated DEGs (Figure 2D; Supplementary Table S3), supporting the conclusion that siSHMT2 only modulated the expression level of a small set of genes. By analyzing the expression pattern of DEGs, we found that they showed obvious difference between siSHMT2 and siNC samples, and were consistent among the three replicates for each group (Figure 2E). Then we analyzed the functions of DEGs by mapping them to the GO and KEGG databases. For up DEGs, they were mapped to regulation of cell morphogenesis, endosome organization, regulation of ventricular cardiac muscle cell membrane repolarization, and cytoplasmic microtubule organization GO pathways (Figure 2F). For down DEGs, we found that they were significantly enriched in regulation of GTPase activity, protein tetramerization, cell migration involved in sprouting angiogenesis, and axon guidance GO pathways (Figure 2G; Supplementary Figure S1B). Finally, we presented several top regulated DEGs, including *PTMA*, *HNRNPR*, *RAPH1*, *TRAF3IP1*, *CNBP*, and *PRR15* (Figure 2H). To confirm the changed expression pattern of DEGs, we performed RT-qPCR experiment for several selected DEGs. We found that the expression levels of *PTMA*, *HNRNPR*, *RAPH1*, and *CNBP* showed consistent and significant changes with the RNA-seq results (Figure 2I). In summary, the DEG results suggest that SHMT2 could affect expression of a small set of genes that were associated with cancer development, indicating a potential novel regulatory mechanism for SHMT2 in HT-1376 cells.

### SHMT2 knockdown modulated the alternative splicing profile of HT-1376 cells

**FIGURE 3:**
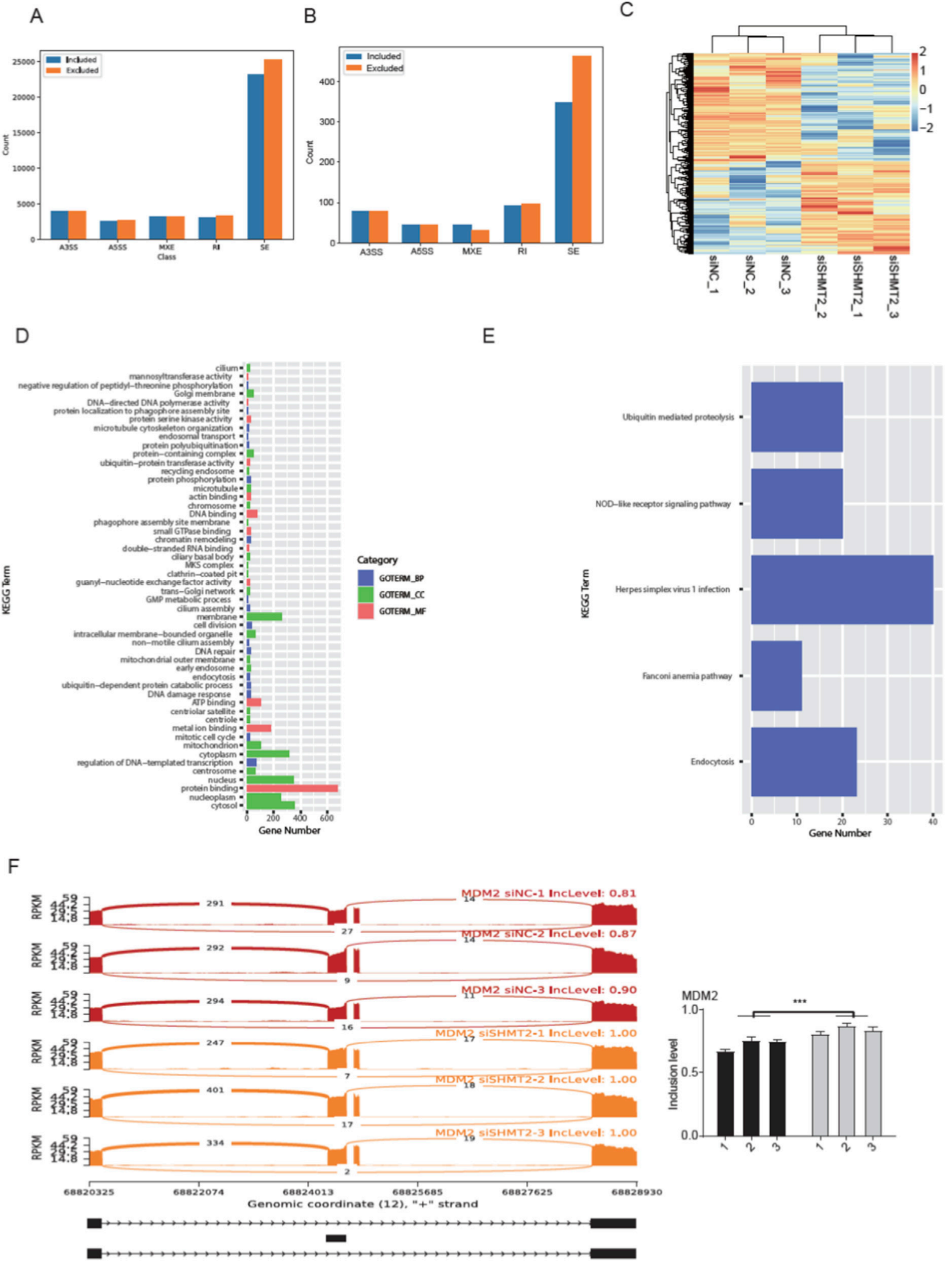
SHMT2 knockdown modulated the alternative splicing profile of HT-1376 cells. **(A)** Bar plot showing the number of all detected ASEs in the six RNA-seq samples. **(B)** Bar plot showing the number of all detected RASEs by siSHMT2 in the six RNA-seq samples. **(C)** Heatmap showing the distribution pattern of ratios for all the RASEs. **(D)** Bar plot showing the top enriched GO pathways for RASGs. **(E)** Bar plot showing the top enriched KEGG pathways for RASGs. **(F)** Reads density and splicing pattern plot for the SE event from MDM2 gene (left panel). Right panel showed the RT-qPCR validation for the SE event.

It has not been reported that SHMT2, as a pyridoxal phosphate-dependent enzyme, can modulate alternative splicing (AS) profile. Meanwhile, AS dysregulation was reported to be associated with carcinogenesis and prognosis of BLCA (Li et al., 2024). Based on the above background, we explored the AS profile regulated by siSHMT2 in HT-1376 cells. We utilized rMATS software (Shen et al., 2014) to identify AS events (ASEs), as well as the SHMT2-regulated AS events (RASEs) between the two groups. Among the detected ASEs, skipped exon (SE) and retained intron (RI) were two dominant ASE types, followed by the alternative 5′ splice site (A5SS), alternative 3′ splice site (A3SS), and mutual exclusive exon (MXE) (Figure 3A; Supplementary Table S4). Then we predicted the RASEs that were significantly regulated between siSHMT2 vs. siNC samples with FDR <0.05 and AS level difference >0.05 as criteria, and identified 1,324 RASEs, including 610 included and 714 excluded RASEs (Figure 3B), indicating that siSHMT2 greatly regulated AS profile of HT-1376 cells. Then we plotted the pattern of RASE ratios and found these RASEs were consistently dysregulated by siSHMT2 in HT-1376 cells (Figure 3C). To further associate the dysregulated AS pattern with SHMT2 functions, we performed functional enrichment analysis for RASGs. GO enrichment analysis demonstrated they were significantly enriched in regulation of DNA−templated transcription, mitotic cell cycle, DNA damage response, DNA repair, cell division, and other pathways (Figure 3D). KEGG analysis reviewed that RASGs were mapped to endocytosis, fanconi anemia pathway, herpes simplex virus one infection, NOD−like receptor signaling pathway, and Ubiquitin mediated proteolysis pathways (Figure 3E). We found that DNA damage and repair pathways were among the top enriched pathways for both GO and KEGG results (Figures 3D,E). We finally presented the dysregulated AS pattern of two RASEs, which were located within MDM2 gene and validated by RT-qPCR experiment (Figure 3F; Supplementary Figure S2). These results indicate that AS dysregulation can probably act as the functional manner for SHMT2 to regulate the development or progression of BLCA.

### SHMT2 knockdown globally altered the metabolism profile of HT-1376 cells

**FIGURE 4:**
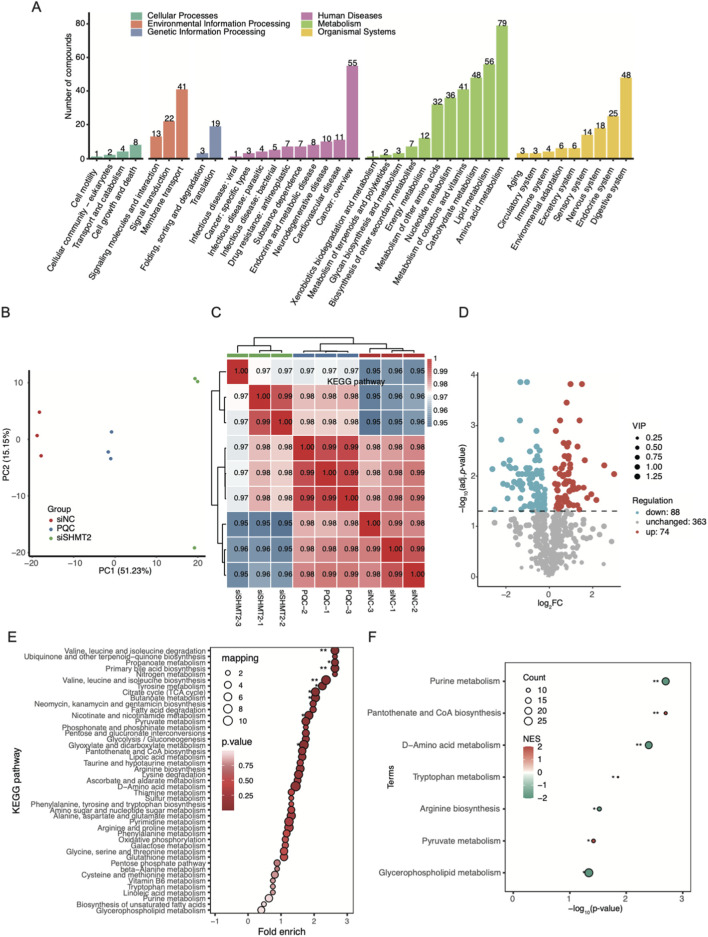
The dysregulated metabolism profile of HT-1376 cells by siSHMT2. **(A)** Bar plot showing the identified KEGG pathways and number of metabolites in each pathway. **(B)** Dot plot showing the clear separation for the siSHMT2, siNC, and PQC samples by PCA results. **(C)** Hierarchical clustering heatmap showing the sample correlation result by Pearson’s correlation coefficient of these three groups. **(D)** Volcano plot showing the upregulated and downregulated DMs between siSHMT2 vs. siNC samples. **(E)** Bubble plot demonstrated the top identified KEGG pathways ranked by fold enrichment. * *p*-value <0.05; ** *p*-value <0.01. **(F)** Bubble plot demonstrated the top enriched KEGG pathways by MSEA method. * *p*-value <0.05; ** *p*-value <0.01.

As an enzyme, SHMT2 has the ability to modulate cellular metabolism to influence signal pathways (Choi et al., 2022). Thus, we collected the siSHMT2 and siNC HT-1376 cells to explore the dysregulated metabolism profile. We used pooled samples as pool quality control (PQC). Finally, we identified 529 metabolites from the six samples. By searching these metabolites in the databases, we performed a simple classification of the detected metabolites to understand the potential functions of metabolites in these samples. We found the metabolism category had the most abundant metabolites by KEGG pathway (Figure 4A). Then we performed PCA for all the detected metabolites and found obvious separation between siSHMT2 and siNC samples (Figure 4B). Pearson’s correlation analysis also demonstrated the clear separation for these three groups (Figure 4C), suggesting that siSHMT2 globally regulated metabolism profile of HT-1376 cells. Next, we performed differential metabolites (DMs) analysis between these two groups, and identified 74 upregulated and 88 downregulated metabolites (Figure 4D), and classified these DMs into six KEGG pathway categories, with amino acid metabolism having the highest number (Supplementary Figure S3). We further explored the enriched KEGG pathways for the DMs and found that valine, leucine and isoleucine biosynthesis and degradation, Primary bile acid biosynthesis, Citrate cycle (TCA cycle), and other pathways were significantly enriched (Figure 4E). Metabolite set enrichment analysis (MSEA) also demonstrated the enrichment of several metabolite sets, including the purine metabolism, Pantothenate and CoA biosynthesis, and D−Amino acid metabolism (Figure 4F). In summary, these results confirmed that SHMT2 can modulate the progression of BLCA probably by modulating the metabolism profile.

## Discussion

The most common tissue type of bladder cancer is the bladder urothelial carcinoma, accounting for about 90%. According to the invasive characteristics, it can be divided into bladder cancer with muscle invasion (75%) and bladder cancer with non-muscle invasion (25%) (Lenis et al., 2020). It is essential to decipher the underlying mechanisms how bladder cancer develops and progresses. In this study, we indicated to decipher the cellular and molecular functions of SHMT2 in BLCA cells and found it can significantly modulate the cell fate, as well as the transcriptome and metabolism profiles. Briefly, we found that SHMT2 knockdown significantly repressed the proliferation rate and altered the cell cycle stages of HT-1376 cells, illustrating the important functions of SHMT2. More importantly, the transcriptome and metabolism profiles identified the downstream targets of SHMT2, helping us deeply understand the regulatory mechanisms of SHMT2. In summary, our study demonstrated how SHMT2 functions in bladder cancer cells and suggested its potential molecular targets, which can be served as valuable therapeutic targets for bladder cancer in future.

Existing research reports have demonstrated through molecular and cellular experiments that SHMT2 is involved in regulating signaling pathways such as cell cycle, redox, apoptosis, and ECM, which affect the proliferation and apoptosis of cancer cells (Zeng et al., 2021; Lee et al., 2014). According to the analysis from multiple databases such as TCGA, SHMT2 is highly expressed in various cancers and is significantly associated with poor prognosis in BLCA. There are studies reporting that succinylation of SHMT2 at the K280 site inhibits the growth of colorectal cancer cells (Yang et al., 2018). In terms of metabolism, SHMT2 affects nucleotide synthesis, which in turn affects cell proliferation. Meanwhile, SHMT2 affects the growth, migration and apoptosis of bladder cancer cells *in vitro*. Simultaneously affecting the expression levels of E-cadherin and N-cadherin, as well as the EMT process, SHMT2 deficiency induces cell apoptosis, ultimately affecting the progression of BLCA. It has been reported that SHMT2 can promote inflammatory cytokine signaling by interacting with the deubiquitylating BRCC36 isopeptidase complex (BRISC), which may be associated with the metabolism function of SHMT2 (Walden et al., 2019). However, its interaction with downstream targets and the molecular mechanisms underlying the regulation of related signaling pathways have not been thoroughly studied in BLCA.

Very few studies have investigated the transcriptome influence of SHMT2. In this study, we first identified the DEGs regulated by siSHMT2. Based on the PCA result, the magnitude of the global transcriptome change by siSHMT2 is not profound, suggesting that SHMT2 did not substantially change the transcriptome profile. Meanwhile, we also detected several important DEGs, including *PTMA*, *HNRNPR*, *RAPH1*, *TRAF3IP1*, *CNBP*, and *PRR15*. PTMA was among the most significant DEGs. Previous study demonstrated that PTMA was significantly upregulated in bladder tumors, and its expression showed a positive correlation with the high grade of bladder cancer (Tsai et al., 2009). HNRNPR, a member of the spliceosome C complex, has been reported to have oncogenic role in human tumors (Yang et al., 2023), and mediate the metastasis of gastric cancer and hepatocellular carcinoma (Chen et al., 2019; Wang et al., 2024). PRR15, which was downregulated in siSHMT2 samples, is a negative regulator for the malignant progression and a clinical prognosis predictor of breast cancer by modulating the PI3K/Akt signaling (Guo et al., 2023). Interestingly, the downregulated gene CNBP by siSHMT2 can also control the tumor cell biology by regulating the expression of tumor-promoting genes by acting as a transcription factor (Lee et al., 2019). In summary, our results demonstrated that SHMT2 can modulate the phenotypes of HT-1376 cells probably by regulating the expression of these genes, which need to be further validated in BLCA.

Another important issue in this study was the globally dysregulated alternative splicing profile by siSHMT2 in HT-1376 cells, which has not been reported before. Among the RASEs, skipped exon was the dominant event. Alternative splicing is dysregulated during BLCA pathogenesis and development and can be novel biomarker for cancer diagnosis, prognosis, and treatment (Li et al., 2024; Montero-Hidalgo et al., 2023). Among the enriched pathways for RASGs, autophagy was outstanding and its modulation is associated with the development and treatment of BLCA (Li et al., 2019). Meanwhile, the enriched KEGG pathway endocytosis is an emerging feature of cancer and a pivotal pathway for regulating metastasis (Khan and Steeg, 2021), indicating that the AS regulation is a promising regulatory manner of SHMT2 in BLCA progression. At the same time, we only checked the AS profile and have not further explored the mechanisms and functions of the dysregulated AS profile, which we will conduct these issues in future studies.

Finally, we identified the differential metabolites in siSHMT2 samples, which reflects the dominant function of SHMT2. Besides the canonical purine metabolism by SHMT2 (Zeng et al., 2021), Among the top enriched metabolism pathways, the amino acid metabolism pathways were detected, including the degradation and biosynthesis of isoleucine, leucine and valine, which belong to the branched-chain amino acids (BCAAs). The metabolism of amino acids has been associated with cancer and can contribute to epigenetic regulation and immune responses linked to tumorigenesis and metastasis (Lieu et al., 2020). In bladder carcinogenesis, leucine and isoleucine have been evaluated their effects and tumor-promoting activity in rats (Gi and Wanibuchi, 2015). Meanwhile, the BCAAs can contribute to cancer progression probably by the modulation of the mTOR activity (Jung et al., 2021). However, previous studies have not investigated the influence of SHMT2 on BCAAs, indicating the novelty of this discovery. Some other pathways, including the Pantothenate and CoA biosynthesis and TCA cycle, have also been linked to the pathogenesis or development of cancers (Eniafe and Jiang, 2021; St Paul et al., 2021).

In conclusion, our study highlights the important regulatory functions of SHMT2 in BLCA HT-1376 cells, and identified the downstream targets and substrates of SHMT2 that may play important roles in the development of BLCA, yielding the novel regulatory manners of SHMT2. However, there are some shortages of this study. It should be better that molecular targets and regulatory mechanisms of SHMT2 are confirmed using other experimental methods, including rescue experiments, CRISPR knockout, pathway inhibition model, or spatial transcriptome technologies (Fan et al., 2025; Fan et al., 2024; Liu et al., 2023). Meanwhile, the RNA-seq and metabolomics may introduce potential biases, thus the results should be confirmed using other methods. And the validation experiments in multiple cell lines or animal models are encouraged to support the conclusions further extend the discoveries in this study. In summary, we propose that SHMT2 has important functions in BLCA by mediating the levels of multiple layer molecules, which can be served as potential therapeutic targets for BLCA in future.

## Data Availability

The datasets presented in this study can be found in online repositories. The raw sequencing data has been deposited in the Gene Expression Omnibus (GEO) database with accession number GSE290189.
