# Supplementary material for: SHMT2 modulates the transcriptome and metabolism profiles to promote the tumor phenotypes of bladder cancer HT-1376 cells
Source: Front Genet. 2025 Nov 20;16:1694089. doi: 10.3389/fgene.2025.1694089 (PMC12674598; doi:10.3389/fgene.2025.1694089)
Supplement: Supplementary file 3 [file Table1.docx]

**Table S1. Primer sequences for PCR experiment.**

| HNRNPR-F: | CCTCCAGATTACTACGGCTATG |
| --- | --- |
| HNRNPR-R: | TTCCTCCTCCTCTTCCTCTTAC |
| RAPH1-F: | CCTGTTAGCCGACATACATTGA |
| RAPH1-R: | AGCAGCCTCATCCATACTCA |
| PTMA-F: | GCGTTCTCTGTCCTACTTCTG |
| PTMA-R: | ACTGGAATGCTCGGAATAAGAT |
| CNBP-F: | AAGTCAACTGTTACCGCTGTG |
| CNBP-R: | TGCCTCTATCTGCCAACCTT |
| MDM2-F: | TGCCTCAGCCTTCCAAGTAA |
| MDM2-R: | GGTGCCTCACATCTGTAATCC |
| GAPDH-F | GGTCGGAGTCAACGGATTTG |
| GAPDH-R | GGAAGATGGTGATGGGATTTC |
