# Supplementary material for: SHMT2 modulates the transcriptome and metabolism profiles to promote the tumor phenotypes of bladder cancer HT-1376 cells
Source: Front Genet. 2025 Nov 20;16:1694089. doi: 10.3389/fgene.2025.1694089 (PMC12674598; doi:10.3389/fgene.2025.1694089)
Supplement: Supplementary file 5 [file DataSheet1.docx]

**Supplementary Figures**


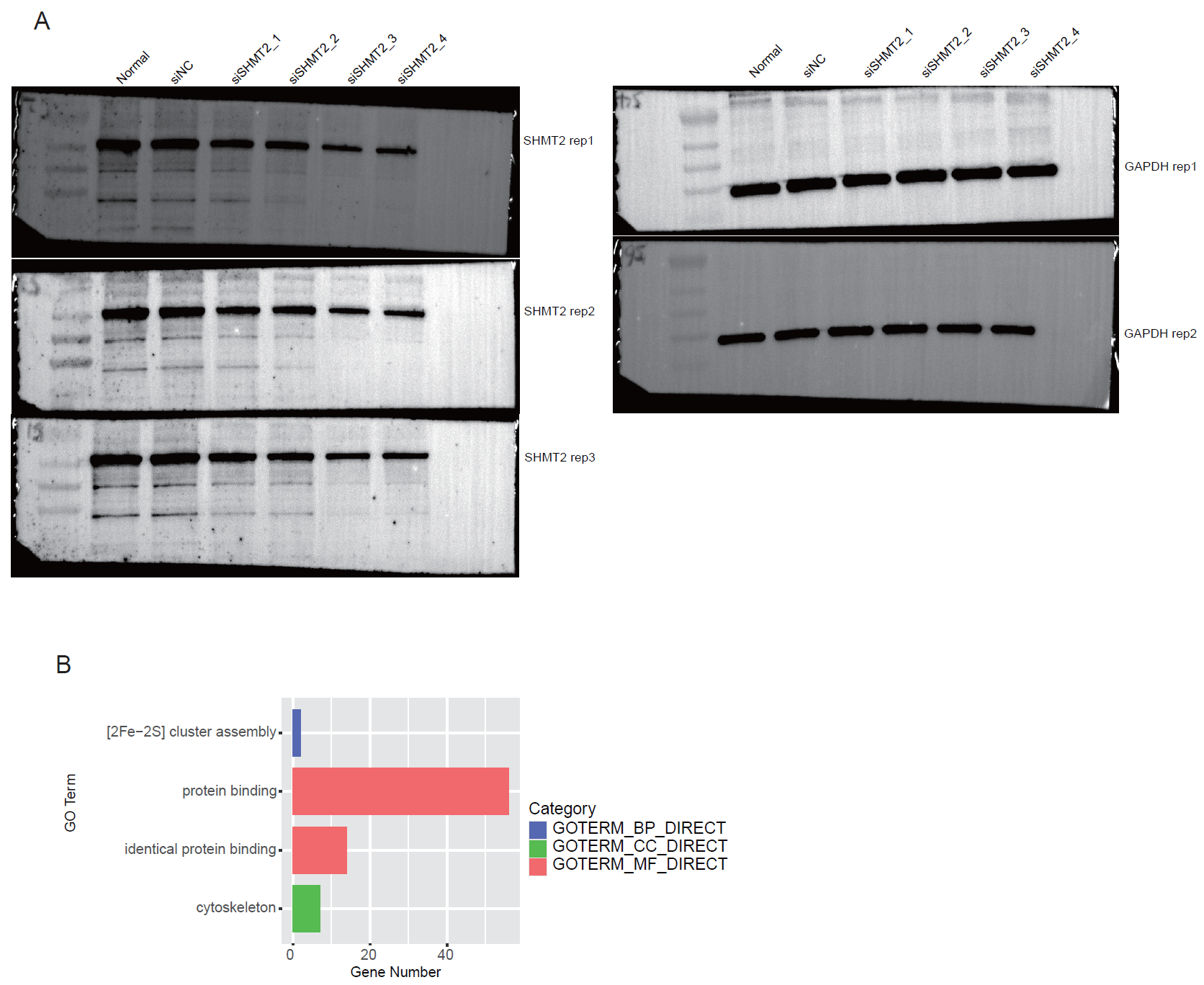


**Figure S1.** Functional analysis of dysregulated transcriptome profile by siSHMT2 in HT-1376 cells. **A.** Western blot result of siSHMT2 in HT-1376 cells. The raw gel plot was shown. **B.** Bar plot showing the GO pathways of DEGs.

**
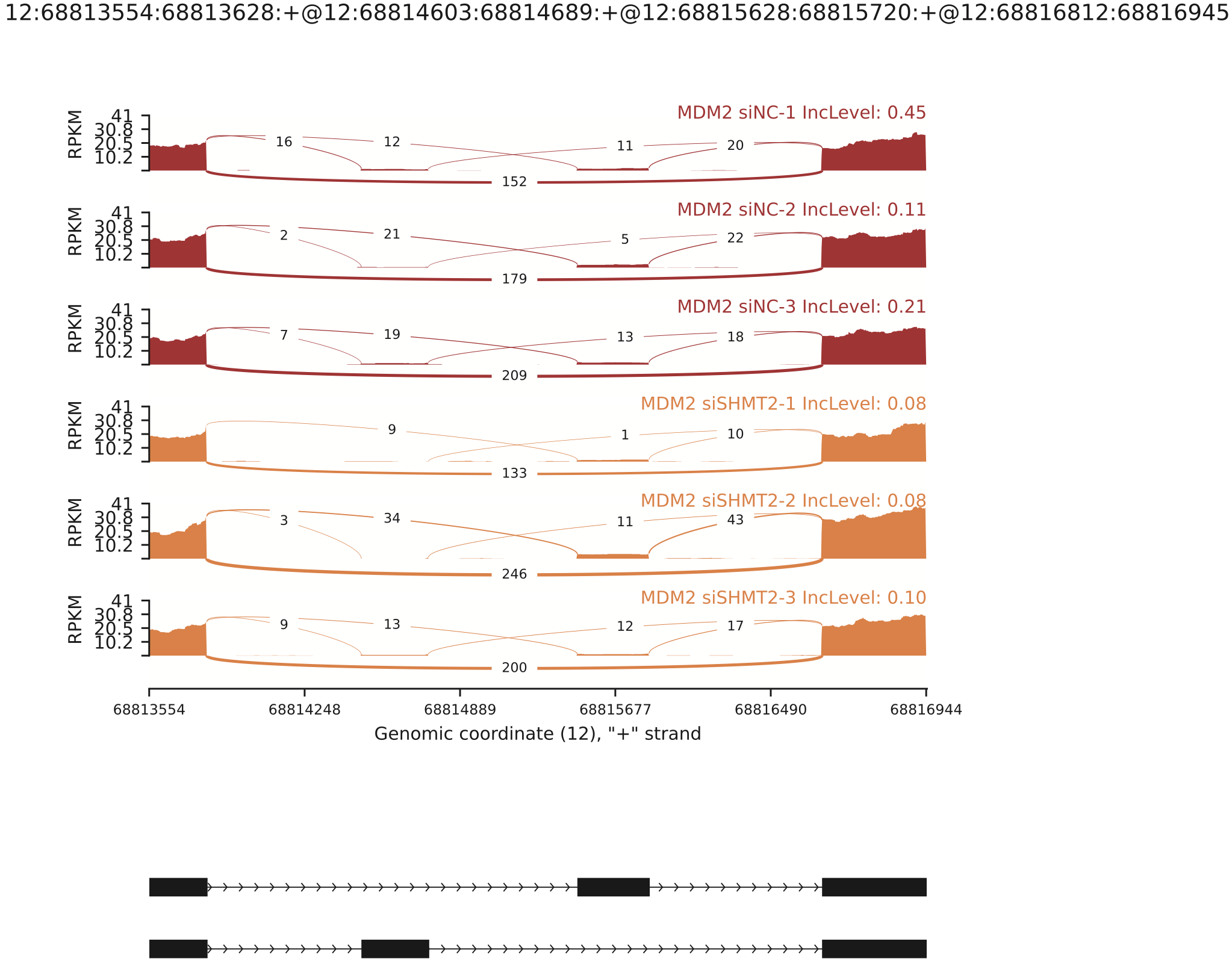
**

**Figure S2.** Reads density and splicing pattern plot for another SE event from MDM2 gene.

**
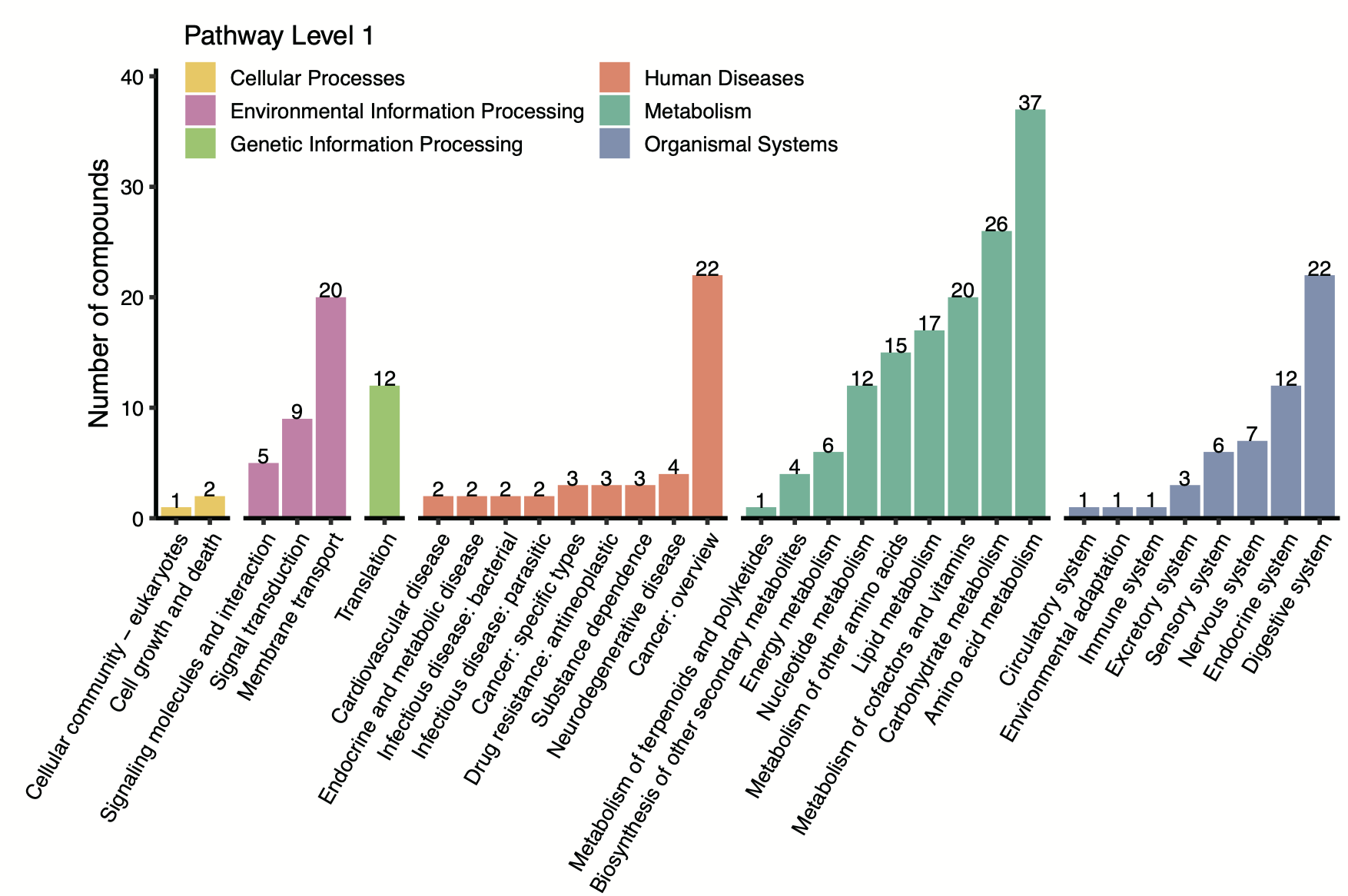
**

**Figure S3.** Bar plot showing the enriched KEGG pathways for all the detected metabolisms.
